# A systematic evaluation of left ventricular (LV) models for estimating LV volumes in children using cardiac cine (MRI)

**DOI:** 10.1186/1532-429X-15-S1-P234

**Published:** 2013-01-30

**Authors:** Jiming Zhang, Carlo Uribe, Jason Liu, Benjamin Cheong, Amol Pednekar, Paolo Angelini, Raja Muthupillai

**Affiliations:** 1 Physcs and Texas Center for Superconductivity, University of Houston, Houston, TX, USA; 2 Center for coronary artery anomalies, Texas Heart Institute, Houston, TX, USA; 3 Glenda Dawson High School, Pearland, TX, USA; 4 Diagnostic and Interventional Radiology, St. Luke's Episcopal Hospital, Houston, TX, USA

## Background

Assumptions about LV shape are often used to estimate LV volumes in ultrasound and x-ray angiography, which are not necessary for CMRI.

### Purpose

Using CMRI as the gold standard, we sought to systematically evaluate the performance of commonly used models, and a linear combination of the models, to estimate LV volumes of children.

## Methods

### Subjects

198 children (137 male, age: 12.4 ± 1.2 years, range 11~15 years) who provided written informed consent, in the context of a screening study for causes of sudden cardiac death, were enrolled in this IRB approved study.

### MRI Acquisition

Breath held CMRI cine images were acquired in standard orientations (4-chamber (4Ch), Left-Ventricular Outflow Tract (LVOT), and Short Axis (Sax)) were acquired using a vendor provided stock SSFP sequence at 1.5 T (Philips Healthcare) with an in-plane resolution < 2 x 2 mm^2^, and an acquired temporal resolution < 50 ms for all subjects.

### Data Analysis

An expert observer drew contours in end-diastole (ED) and in end-systole (ES) to estimate LV volumes on CMR images acquired in all orientations (Sax, LVOT, and 4Ch). In 4Ch and LVOT views, longitudinal length (distance from mitral valve annulus to apical endocardium), and the transverse width (at mitral valve annulus) of the LV were recorded.

### Models

**Figure 1:**
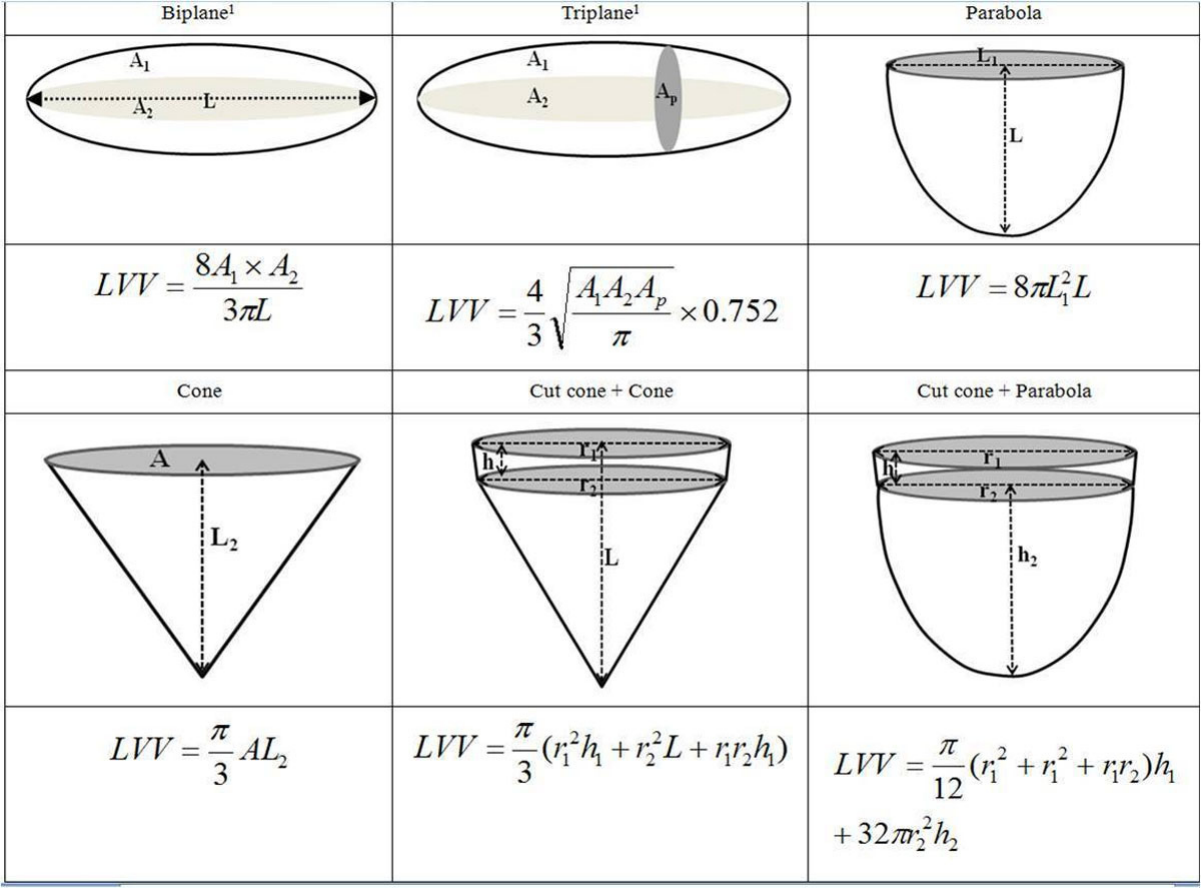
Geometric models considered in the current study. A, L_1_ and r_2_: area, diameter and radius from short-axis slice at papillary muscle level; L: longer length from apex to base in 4CH and LVOT views; r_1_: bigger half width at mitral valve annulus in 4CH and LVOT views; h_1_: length from base to papillary muscle level; h_2_ = L-h_1_

The performance of six geometric models (described in Figure 1), and 15 weighted models generated (LVV_w_) using a linear combination of any two of the six geometric models (LVV_w_ = α_i_LVV_i_+β_j_LVV_j_, where α_i_, β_j_ were the coefficients estimated in 100 subjects via minimizing the least-square's error compared to volumes estimated from Sax) were evaluated.

### Data Analysis

Using Sax volumes as the reference, percentage-error of EDV, ESV, and ejection fraction (EF) of all 21 models were calculated.

## Results

Commonly used biplane[1] and triplane[1] ellipsoid models have significant mean percentage error (at least 9%) for EDV/ESV/EF (Table 1), and triplane model was more reproducible than bi-plane model. A linear-weighted model of biplane and parabola substantially diminishes percentage error for EDV, ESV and EF to 0.3±6.0%, 0.5±10.7% and 0.0±6.4% (α_EDV_ = 0.4, β_EDV_=0.7; α_ESV_ = 0.9, β_ESV_=0.2) respectively.

**Table 1:** Estimation result of EDV, ESV and EF by weighted models

[%]		Biplane	Triplane	Parabola	Cone	Cutcone+Parabola	Cutcone+Cone
	EDV	10.4±9.8	0.3±6.0	**0.3±6.0**	0.3±6.0	-0.7±7.0	-1.0±7.4
	
Biplane	ESV	-10.8±16.0	0.5±11.0	**0.5±10.7**	0.5±10.7	-0.7±7.0	-1.3±11.8
	
	EF	13.3±8.6	0.0±6.5	**0.0±6.4**	0.0±6.4	0.1±6.7	0.2±7.3

	EDV	X	-16.1±5.1	0.3±6.0	0.3±6.0	0.0±6.1	-0.1±6.1
	
Triplane	ESV	X	-27.2±8.7	0.5±10.7	0.5±10.7	-0.4±10.4	-0.6±10.8
	
	EF	X	9.2±6.4	0.0±6.5	0.0±6.5	0.4±6.6	0.5±6.7

	EDV	X	X	-14.0±6.6	0.7±7.7	0.2±7.6	0.2±7.6
	
Parabola	ESV	X	X	-19.5±9.1	0.3±11.4	-0.4±10.6	-0.5±10.8
	
	EF	X	X	4.4±7.1	0.3±7.4	0.5±7.0	0.5±7.2

	EDV	X	X	X	-43.4±4.3	0.2±7.6	0.2±7.6
	
Cone	ESV	X	X	X	-47.0±6.0	-0.4±10.6	-0.5±10.8
	
	EF	X	X	X	4.4±7.1	0.5±7.0	0.0±8.8

	EDV	X	X	X	X	14.0±11.2	0.0±8.8
	
Cutcone+Parabola	ESV	X	X	X	X	15.3±13.4	-0.1±10.3
	
	EF	X	X	X	X	0.9±7.9	0.1±6.9

	EDV	X	X	X	X	X	-1.6±11.1
	
Cutcone+Cone	ESV	X	X	X	X	X	0.3±13.5
	
	EF	X	X	X	X	X	-1.6±9.3

## Conclusions

In this group of young children, our results show that the estimation of LV volumes of conventional bi-plane and triplane models can be significantly improved by using a linear weighted model of bi-plane and parabolic models. These findings have to be confirmed in a larger study.

## Funding

The study was partly funded by Texas Heart Institue.
